# Anionic Water Cluster Polymers [(H_2_O)_18_(OH)_2_]*_n_*^2*n*−^ Is Stabilized by Bis(2,2′-bipyridine) Cupric Chloride [Cu(bipy)_2_Cl]^−^

**DOI:** 10.3390/molecules23010195

**Published:** 2018-01-19

**Authors:** E Liu, Fangfang Jian

**Affiliations:** School of Chemical Engineering and Pharmaceutics, Henan University of Science and Technology, Luoyang 471023, China; 798233143@haust.edu.cn

**Keywords:** water cluster, crystal, hydrogen-bond, polymer, Cu compound

## Abstract

Anionic water clusters have long been studied to infer properties of the bulk hydrated electron. In particular, the question of whether the excess electron is on the surface of the cluster or in the interior of the clusters has been the subject of much speculation. The successes of solid-state physics are built on exploiting the regularity of atomic arrangements in crystal. Describing the crystalline order of solids is relatively straightforward. Here we report the crystal structure of an anionic water cluster polymer [(H_2_O)_18_(OH)_2_]*_n_*^2*n*−^ moiety that is stabilized by bis(2,2′-bipyridine) cupric chloride [Cu(bipy)_2_Cl]^−^ host.

## 1. Introduction

Studying water clusters can offer insight into the properties of water in various environments, and clusters have played a major role in theoretical approaches to understanding the properties of liquid water and ice [1,2,3]. There has been significant attention given to the understanding of ordered water clusters in hydrophobic environments because of their importance in chemical and biological interfaces [4,5]. Structural studies of water clusters within the lattice of a crystal host have significantly advanced our knowledge toward understanding the behavior of bulk water [6,7,8,9,10]. Structural information of the water clusters is the first step toward understanding the behavior of bulk water [11,12,13,14]. Hydrogen-bonded water networks can grow naturally at the hydrophobic surface, and often give rise to a stable hydrate [15,16,17,18].

**Scheme 1:**
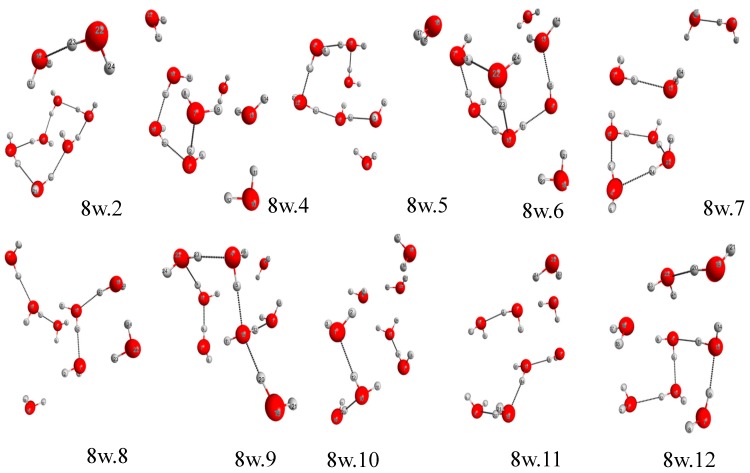
Geometries of the anionic water cluster (H_2_O)_8_, predicted theoretically by Peng Xu and Mark S. Gordon.

The anion water clusters of X^−^(H_2_O)*_n_* have been extensively investigated both experimentally and theoretically due to their suitable simplified model systems for aerosol study and molecular recognition study to design the X receptors [19,20,21]. In anion water clusters of X^−^(H_2_O)*_n_*, the hydroxyl anion water cluster is of particular interest because the hydroxide ion (OH^−^) and hydrogen ion (H^+^) are the two essential ionic species in aqueous chemistry. Since the first anionic water cluster, OH^−^(H_2_O)*_n_* [22], was found by mass spectrometry, a probing of water cluster ions using the various vibrational spectroscopies and quantum chemistry calculations has been done [23,24,25,26,27]. The discussed topics in hydroxyl hydration are whether the hydroxyl is inside the clusters or on their surface and how many water molecules are necessary for its complete solvation [28]. In addition, various models for the (H_2_O)^−^*_n_* anion cluster have been theoretically predicted [29] (Scheme 1, *n* = 8). However, precise crystal data for the (H_2_O)^−^*_n_* anion cluster, to our knowledge, are very limited [30,31]. Since the distinction of the structural variation of the anionic water cluster is beyond the scope of vibrational spectroscopic precision, we think that crystallographic structural studies of the anion water cluster stabilized in the lattices of crystal hosts, rather than the spectroscopic investigations, should provide much more detailed information characterizing these intriguing and important clusters. In this paper, we report the crystal structure data of compound [CuCl(bipy)_2_]_2_[(OH)_2_(H_2_O)_11_] (1), and find anionic water cluster polymer [(H_2_O)_18_(OH)_2_]*_n_*^2*n*−^ is stabilized by bis(2,2′-bipyridine) cupric chloride [Cu(bipy)_2_Cl]^−^. From the crystal structure of anionic water cluster complex to be obtained, we can get two important pieces of structure information: (1) the excess electron is trapped in the water cluster interior; (2) oxygen atoms, linked by H-bonds to acceptors and donors, can form various geometry fashions as carbon sp^3^ covalent chemistry. Also, as with covalent bonds, the single bond, and double bond oxygen-oxygen can be suggested.

## 2. Results

Add 2,2′-bipyridine to the fresh precipitation Cu(OH)_2_ water solution, and stir the solution for three hours at about 60 °C. After filtering out the precipitate, the resulting solution was left to stand undisturbed. Upon slow evaporation at room temperature, deep blue crystals were obtained from the mother liquor. They were all collected, dried, and submitted for elemental analysis. X-ray crystallographic study has confirmed the existence of [CuCl(bipy)_2_]_2_[(OH)_2_(H_2_O)_11_] (**1**).

**Figure 1:**
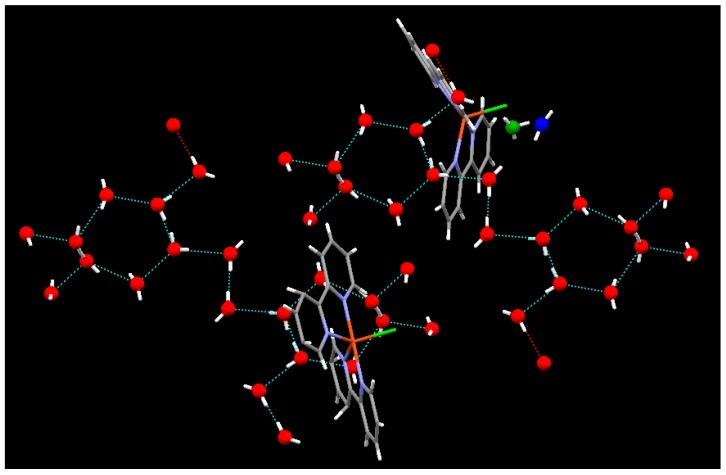
Packing of anion water clusters [(H_2_O)_18_(OH)_2_]*_n_*^2*n*−^and three H_2_O and the cation [CuCl(bipy)_2_]^+^ moiety along the *pc* plane. Red ball represent water cluster, blue ball is O4w, and green ball is O5w.

**Figure 2:**
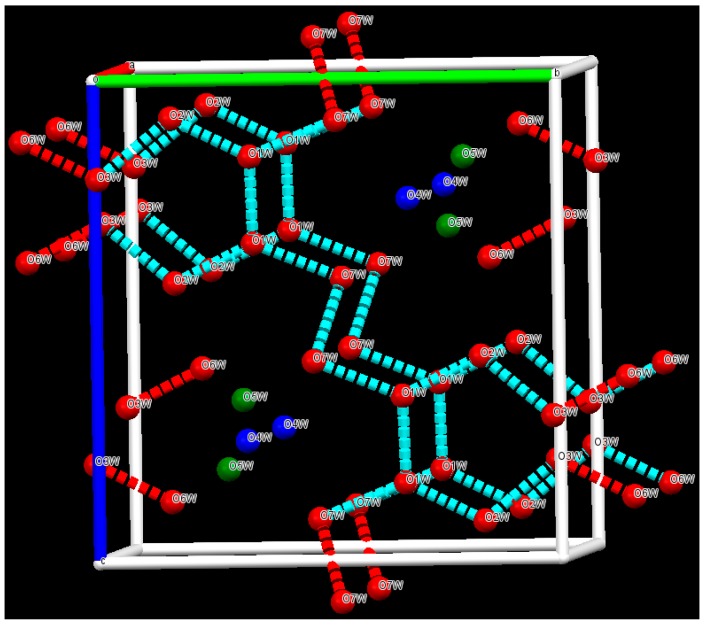
View of the anion water cluster polymer [(H_2_O)_18_(OH)_2_]*_n_*^2*n*−^ along the *pc* plane, which is like *trans*-1,6-poly(3,4-dimethyl phenylethylene) structure. O4w and O5w are the outside of water cluster polymer.

**Figure 3:**
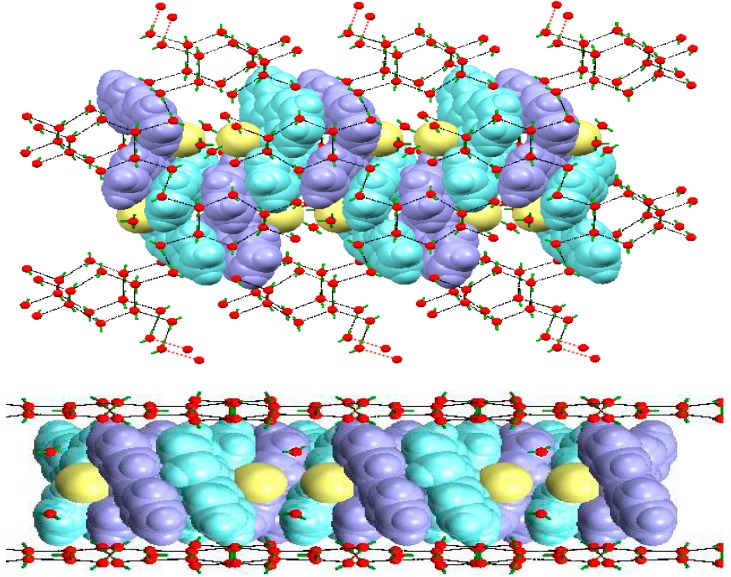
View along the *a* axis (**Top**) and *c* axis (**Bottom**) showing the host [CuCl(bipy)_2_]^+^ cations sandwiched between two nearly planar water cluster sheet.

Figure 1 shows the packing of anion water cluster polymers [(H_2_O)_18_(OH)_2_]*_n_*^2*n*−^ is stabilized by the cation [CuCl(bipy)_2_]^+^ moiety along the *bc* plane. Figure 2 shows the perspective diagram of [(OH)_2_(H_2_O)_18_]^2−^ anionic water cluster viewed along the *bc* plane for compound (**1**). The (H_2_O)_11_ water molecules are divided into two groups. One is water cluster polymer [(H_2_O)_18_]*_n_* connected with two OH^−^ anions to form “*trans*-1,6-poly(3,4-dimethyl phenylethylene) structure-shaped”. The other is water molecules to form (H_2_O)_4_ monomer through O···O hydrogen bonds. Figure 3 shows the view along the *a* axis (Top) and *c* axis (Bottom) showing the host [CuCl(bipy)_2_]^+^ cations sandwiched between two nearly planar water cluster sheet. Table 1 and Table 2 list the hydrogen bonds and π-π interactions, respectively.

## 3. Discussion

The structure unit of the compound (**1**) contains a pair of independent cations [CuCl(bipy)_2_]^+^, anionic water cluster polymer [(H_2_O)_18_(OH)_2_]*_n_*^2*n*−^ and four water molecules (Figure 1). Only the electrostatic interactions between cations [CuCl(bipy)_2_]^+^ and anionic water cluster polymer [(H_2_O)_18_(OH)_2_]*_n_*^2*n*−^ govern the binding in the system. There are no obvious bonding interactions between the two independent [CuCl(bipy)_2_]^+^ cations, with the distance of adjacent Cu(1)∙∙∙Cu(2) 5.531(1) Å. The coordination environments of two Cu(II) atoms are similar. Each Cu(II) atom is pentacoordinated with four N atoms from two 2,2′-bipyridine ligands and one Cl atom in a distorted square pyramidal geometry. Four nitrogen atoms from two bipyridine occupy the basal sites with Cu-N distances of 1.879(5) and 2.430(6) Å for Cu(1), 1.761(5) and 2.446(7) Å for Cu(2). The coordinated chloride occupies the apical position with Cu(1)-Cl(1) distances of 2.281(3) Å and Cu(2)-Cl(2) 2.284(3) Å, respectively. The bite angle of chelating bipyridine ligands N(1)-Cu(1)-N(2) and N(3)-Cu(2)-N(4) is 82.9(2)° and 82.8(3)°, respectively. The maximum deviation of 0.095(3) Å of Cu(1) and 0.084(3) Å of Cu(2) atom from the mean plane calculated from 13 atoms of Cu and bipyridine ligands shows that they are all almost planar. In the crystal building, the cationic complexes hold together by means of face-to-face and edge-to-face *π*-*π* interactions among the aromatic bipyridine ligands to form layers parallel to the *ab* plane (Table 2).

It is especially interesting to note that eighteen water molecules and two hydroxyl groups in compound (**1**) form anionic water cluster polymer [(H_2_O)_18_(OH)_2_]*_n_*^2*n*−^, like “*trans*-1,6-poly (3,4-dimethyl phenylethylene) structure-shaped” along the *bc* plane, and two O4w and two O5w are the outside of water cluster polymer, which is shown in Figure 2. Though the X-ray structure is not refined at a level that can isolate the position of the hydrogens, and it cannot identify the position of the hydroxyl groups, the charge neutrality requires two of thirteen water molecules to be anions. Of course, considering the symmetrical operation and electric charge balance, O4w is not anions. If O5w was anion, it should not be reasonable because of two anions distance of 3.454 Å for O5w∙∙∙Cl(1) and 3.602 Å for O5w–Cl(2), respectively. Hence, only the oxygen atom in the water cluster units should be anion. According to the crystal data, we think O6 is hydroxyl anion groups. The water cluster [(H_2_O)_8_(OH)_2_]*_n_*^2*n*−^ units are strongly held together by O-H∙∙∙O interactions with the O∙∙∙O distance ranging from 1.947 to 2.975 Å (average: 2.721 Å) and the O∙∙∙O∙∙∙O angle from 93.03° to 115.28° (average: 109.33°). These values are close to the corresponding values in ice *I_h_*. Furthermore, this aggregate is formally T6(0)A2 plu C2, which has not been introduced in references [32]. The three water molecules O1w, O2w and O3w on the C2 symmetry create a hexamer unit, and the two coterminous O7w in a *tran* mode are linked to O1w molecules in the hexamer unit. The negative charge hydroxyl group (O6) with O3w forms strong hydrogen bond, and the donor/acceptor distance of 2.626 Å. The O3w is obviously deviated from the plane comprising O1w and O2w, resulting in a quasiplanar geometry of the hexamer unit. The O3w∙∙∙O3w distance of 1.947 Å is less than 2.0 Å, which is think a disordered at first. We have resolved the crystal structure according to O3w disordered and the occupancies of 20%, 50% and 80%, respectively, and found the crystal data no good. Therefore we think perhaps negative charge OH^−^ is the key to effecting the O3w water molecules. On the other hand, the interesting structure phenomenon is that water cluster [(H_2_O)_18_(OH)_2_]*_n_*^2*n*−^ form much similar geometry configuration to that of *trans*-1,6-poly(3,4-dimethyl phenylethylene, such as there are three longer bonds and three shorter bonds in the hexamer unit, O7w-O7w bond are *tran*-form.

The anionic water cluster polymer [(H_2_O)_18_(OH)_2_]*_n_*^2*n*−^with the host [CuCl(bipy)_2_]^+^ cations form ionic bonds by electrostatic forces. The one-dimensional anionic water cluster polymers, [(H_2_O)_18_(OH)_2_]*_n_*^2*n*−^, alternate each other and form independent two-dimensional water layers running parallel to (100). The distances between the adjacent water layers is approximately 12.344 Å, with the shortest distance of two H atoms being 9.721 Å. The thickness of water layers is about 1.947 Å. Figure 3 shows the perspective view of the structurally two-dimensional anion water cluster layers, which is separated by the host [CuCl(bipy)_2_]^+^ cations, and form the host [CuCl(bipy)_2_]^+^ cations sandwiched between two nearly planar water cluster sheet. In addition, the Cu atoms in interlayer of the host [CuCl(bipy)_2_]^+^ cations are all in the same plane, with the distance of adjacent Cu(1)∙∙∙Cu(2) 5.531(1) Å, Cu(1)∙∙∙Cu(1) or Cu(2)∙∙∙Cu(2) 7.624 Å. The distance of Cu layer-layer is also 12.344 Å (Supplementary Materials Figure S5).

There are some weak intermolecular interactions between the anionic water cluster polymers [(H_2_O)_18_(OH)_2_]*_n_*^2*n*^ and four crystallization water molecules with the distance of O7w∙∙O4w 3.295 Å. The crystallization water molecules O4w locate on the anionic water cluster layer, and O5w water molecules are between the host [CuCl(bipy)_2_]^+^ cations. The two-dimensional anionic water polymer layers along the (100) direction with extended one-dimensional supramolecular assembly of chloride-water cluster along the *c* axis intercross and form ladder-shaped structure (Supplementary Materials Figure S6).

**Figure 4:**
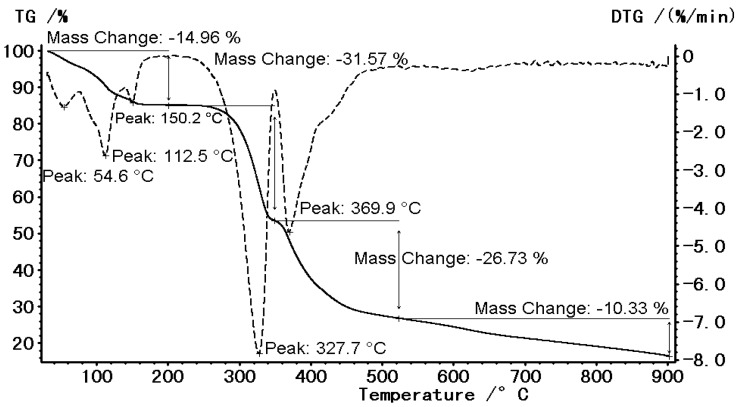
TG and DTG analysis for compound (**1**).

To get more insight into the properties relative to water cluster, the dehydration behavior of compound (**1**) has been investigated using thermogravimetric analysis. The TG and DTG curves of compound (**1**) are shown in Figure 4. The weight loss begins at 54.6 °C, and shows an obvious inflexion at about 150.2 °C. The first step corresponds to the loss of nine water molecules with three heat-absorption peaks (found 14.96% calc. 15.36%). From 150.2 °C to 267.5 °C, there is no weight loss to be observed, indicating the residue [CuCl(bipy)_2_]_2_[(OH)_2_] is very stable. At 327.7 °C, there is an intense endothermic phenomenon, the weight loss of 31.57% correspond to two bipy groups (calc. 29.58%). The residue is [CuCl]_2_(OH)_2_. At about 350 °C, there is a little inflexion, suggesting the residual compound [CuCl]_2_(OH)_2_ is stable. Upon the temperature increases, and there is weight-loss of (26.73% + 10.33%) suggesting the decomposing of [CuCl]_2_(OH)_2_. The final residual compound maybe is CuO (calc. 15.08%, Found 16.11%).

**Figure 5:**
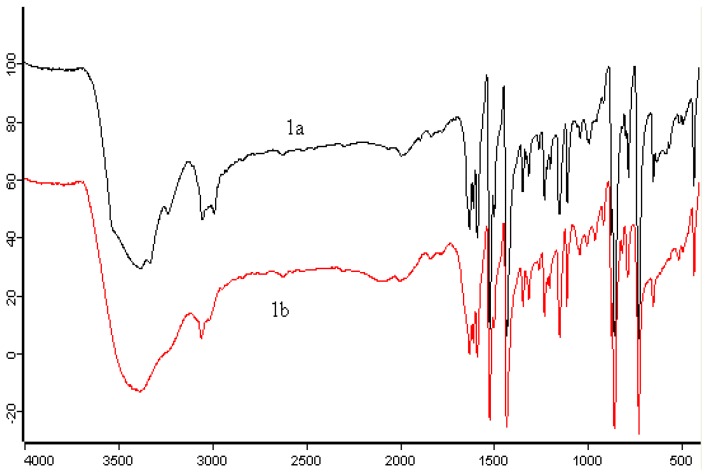
IR spectra of compound (**1**). (**1a**) The original crystalline samples; (**1b**) The residuum at 150.2 °C.

The IR spectrum can also afford the useful structure information to testify the structure of compound (**1**). Figure 5 shows the IR spectrum of original compound (**1a**) and residuum (**1b**) after dehydration at the inflection point temperature 150.2 °C. As shown in Figure 5, the characteristic peaks at 3000 cm^−1^, 3250 cm^−1^ and 3380 cm^−1^ for H_2_O are not observable compared to (**1a**) with (**1b**), indicating that some water molecules are lost. The only unchanged peaks at 3100 cm^−1^ and 3410 cm^−1^ are attributed to hydrogen-bonded OH stretching vibration [33].

## 4. Materials and Methods

The C, H and N elemental analyses were performed on a Perkin-Elmer elemental analyzer. Crystals data were collected on an Enraf-Nonius CAD-4 diffractometer with graphite monochromated Mo *K_α_* radiation (λ = 0.71073 Å). Intensities were corrected for Lorentz and polarization effects and empirical absorption, and the data reduction was carried out. The structure was analyzed by direct method. These data can be observed from the Cambridge Crystallographic Data Center via www.ccdc.cam.ac.uk/data-request/cif. The CCDC number is.

A typical experimental procedure for compound **1** is below: Cupric chloride, sodium hydroxide, 2,2′-bipyridine and other chemical reagents were obtained from commercial sources and used without further purification. To a 100 mL flask 0.01 mol of CuCl_2_∙2H_2_O (1.70 g), 0.01 mol of NaOH (0.40 g) in 40 mL of deionized water, 0.02 mol (3.20 g) of 2,2′-bipyridine in 20 mL of ethanol was added while stirring at temperature 50~60 °C. The reaction was maintained three hours until the solvent was turned to clarify, and then was filtered. The deep blue single crystals suitable for X-ray measurements were obtained by slow evaporation of the resulting solution. Yield: 70% (bases on cupric chloride, CuCl_2_∙2H_2_O). From the element analysis below and the single crystal X-ray, we conclude the compound **1** is [CuCl(bipy)_2_]_2_[(OH)_2_(H_2_O)_11_]. Anal. calc. for C_40_H_56_Cl_2_Cu_2_N_8_O_13_: C, 45.50%; H, 5.31%; N, 10.62%, Cu; 12.04%, Cl, 7.11%; O, 19.72%. Found: C, 45.32%; H, 5.30%; N, 10.51%.

## 5. Conclusions

In summary, we have shown that an anionic water cluster polymer [(H_2_O)_18_(OH)_2_]*_n_*^2*n*−^ is stabilized by bis(2,2′-bipyridine) cupric chloride [Cu(bipy)_2_Cl]^−^. The present aggregate mode, T6(0)A2, has been first reported experimentally. The unique, discrete, “*trans*-1,6-poly(3,4-dimethylphenyl ethylene)” structure-like hydroxyl anion water cluster in compound (**1**) suggests that water molecules may be comparable with carbon *sp*^3^ covalent chemistry, and form various geometry structures such as carbon atoms. The precise structure information of these hydroxyl anion clusters are helpful for improving the modeling of some of the unexplained properties of water and understanding better the structure and behavior of water molecules in chemical and biological process. Also, we reported a simple method to synthesize anion water cluster complexes. Further works to use *o*-phenanthroline etc., neutral ligands or other metal salts are in progress.

## Figures and Tables

**Table 1:** Geometrical Parameters of Hydrogen Bonds (Å, deg) for the Water Cluster.

Length		Angle	
O1w–O2w	2.740(1)	O2w…O1w…O1wA	115.28
O1w–O7w	2.948(1)	O2w…O1w…O7w	130.28
O1w–C(11) ^a^	3.372(1)	O1wA…O1w…O7w	113.35
O1w–C(12) ^a^	3.361(1)	O1w…O2w…O3w	113.25
C(1)–O3w ^a^	3.348(1)	O1w…O2w…O6wB	119.13
C(1)–O2w ^a^	3.386(1)	O3w…O2w…O6wB	125.39
Cl(2)–O5w ^a^	3.454(1)	O3wA…O3w…O6wC	111.78
C(12)–O2w ^a^	3.396(2)	O3wA…O3w…O2w	111.75
C(18)–O7w ^b^	3.264(1)	O6w…O3w…O2w	114.28
O2w–O3w	2.934	O6wD…O4w…O6wE	78.77
O2w–C(2) ^a^	3.386(1)	O6wD…O4w…O7w	166.87
O3w–O6w ^c^	2.625(1)	O6wE…O4w…O7wA	166.87
O5w-O6w ^b^	3.176	O6wE…O4w…O7w	90.53

Symmetry code: ^a^, 1 − x, y, 1/2 − z; ^b^, x, 1 − y, −1/2 + z; ^c^, x, −y, −1/2 + z. A, −x, y, 1/2 − z; B, −x, y, 3/2 − z; C, x, −y, −1/2 + z; D, −x, 1 − y, 1 − z; E, x, 1 − y, −1/2 + z.

**Table 2:** π-π Interactions (Face-to-Face) and C-π Interactions in compound (**1**) ^a^.

Ring (*i*) → Ring (*j*)/C	Distance between the *(i,j*) Ring Centroids (Å)	Dihedral Angle (*i,j*) (Deg)	Distance of Centroid (*i*) from Ring (*j*) (Å)
R1 → R5 ^i^	3.813	2.55	3.268
R1 → R6 ^ii^	4.254	56.05	0.567
R1 → R7 ^iii^	4.287	28.65	4.175
R2 → R5 ^iv^	3.813	2.55	3.268
R2 → R6 ^iii^	4.254	56.05	0.567
R2 → R7 ^ii^	4.287	28.65	4.175
R3 → R6 ^iii^	4.016	28.02	3.982
R4 → R6 ^ii^	4.016	28.02	3.982
R5 → R6 ^i^	3.730	1.96	3.266
R7 → R5 ^iii^	4.088	26.36	3.597
R7 → R8 ^i^	3.783	0.43	3.431
C4 → R1 ^i^	3.251		3.334
C4 → R2 ^v^	3.251		3.334

^a^ Symmetry code: (i) = 1 − x, −y, −z; (ii) = 1 − x, y, 1/2 − z; (iii) = x, y, z; (iv) = x, −y, 1/2 + z; (v) = x, −y, −1/2 + z. R(*i*)/R(*j*) denotes the ith/jth rings of phen: R(1) = Cu(1)/N(1)/C(5)/C(6)/N(2); R(2) = Cu(1)/N(1)a/C(5)a/C(6)a/N(2)a; R(3) = Cu(2)/N(3)/C(15)/C(16)/N(4); R(4) = Cu(2)/N(3)b/C(15)b/C(16)b/N(4)b; R(5) = N(1)/C(1)/C(2)/C(3)/C(4)/C(5); R(6) = N(2)/C(6)/C(7)/C(8)/C(9)/C(10); R(7) = N(3)/C(11)/C(12) /C(13)/C(14)/C(15); R(8) = N(4)/C(16)/C(17)/C(18)/C(19)/C(20).

## Supplementary Materials

The following are available online.
