# Supplementary material for: Anionic Water Cluster Polymers [(H2O)18(OH)2]n2n− Is Stabilized by Bis(2,2′-bipyridine) Cupric Chloride [Cu(bipy)2Cl]−
Source: Molecules. 2018 Jan 19;23(1):195. doi: 10.3390/molecules23010195 (PMC6017829; doi:10.3390/molecules23010195)
Supplement: Supplementary file 1 [file molecules-23-00195-s001.pdf]

Supporting information

Anionic Water Cluster Polymers $[(H_2O)_{18}(OH)_2]_n^{2n-}$ is Stabilized by Bis(2,2'-bipyridine) Cupric Chloride $[Cu(bipy)_2Cl]^-$

*E Liu, Fangfang Jian**

School of Chemical Engineering and Pharmaceutics,
Henan University of Science and Technology, Luoyang 471023, China;
798233143@haust.edu.cn (E.L.)
*Correspondence: ffj2013@163.com; Tel.: +86-138-0895-9914

Table of contents **Page**

1. General **S-1**

2. Perspective View **S-2 ~ S-3**

3. Crystallographic data for $[CuCl(bipy)_2]_2[(OH)_2(H_2O)_{11}]$ **S-4**

4. Table of bond lengths and angles **S-5 ~ S-6**

5. Table of Geometrical Parameters of Hydrogen Bonds for the Water Cluster **S-7**

6. Table of *π-π* Interactions (Face-to-Face) and C-*π* Interactions **S-8**

7. Schematic view of the Cu-layers of the 2D network. **S-9~S-10**

8. CIF file for $[CuCl(bipy)_2]_2[(OH)_2(H_2O)_{11}]$ **S11~S21**

1. General

The C, Hand N elemental analyses were performed on a Perkin-Elmer elemental analyzer. Crystals data were collected on an Enraf-Nonius CAD-4 diffractometer with graphite monochromated Mo K_α radiation (λ = 0.71073Å). Intensities were corrected for Lorentz and polarization effects and empirical absorption, and the data reduction was carried out using SADABS program. The structure was solved by direct methods using SHELXS-97. All the non-hydrogen atoms were refined on F^2 anisotropically by full-matrix least squares method. The hydrogen atom positions were fixed geometrically at calculated distances and allowed to ride on the parent carbon atoms. Atomic scattering factors and anomalous dispersion corrections were taken from International Table for X-Ray Crystallography. Summaries of crystal and intensity collection, and bond distances and angles of the compounds are given.

A typical experimental procedure for compounds **1** are below: Cupric chloride, sodium hydroxide, glycyl glycine, 2,2'-bipyridine and other chemical reagents were obtained from commercial sources and used without further purification. To a 100 mL flask 0.01 mol of $CuCl_2·2H_2O$ (1.70 g), 0.01 mol of NaOH (0.40 g) in 40 mL of deionized water, 0.02 mol (3.20 g) of 2,2'-bipyridine in 20 mL of ethanol was added with stirring at temperature 50 ~ 60°C. The reaction was maintained three hours until the solvent was turned to clarify, and then was filtered. The deep blue single crystals suitable for X-ray measurements were obtained by slow evaporation of the resulting solution. Yield: 70% (bases on cupric chloride, $CuCl_2·2H_2O$). From the element analysis below and the single crystal X-ray, we conclude the compound **1** is $[CuCl(bipy)_2]_2[(OH)_2(H_2O)_{11}]$. Anal. Calc. for $C_{40}H_{56}Cl_2Cu_2N_8O_{13}$: C, 45.50%; H, 5.31%; N, 10.62%, Cu; 12.04%, Cl, 7.11%; O, 19.72%. Found: C, 45.32%; H, 5.30%; N, 10.51%.

2. Perspective View

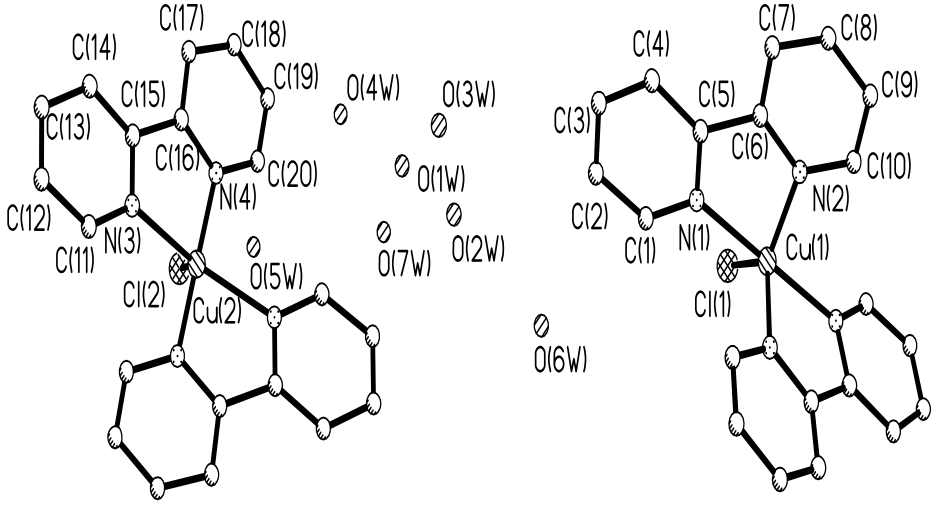

Fig. S1. ORTEP drawing of structural unit

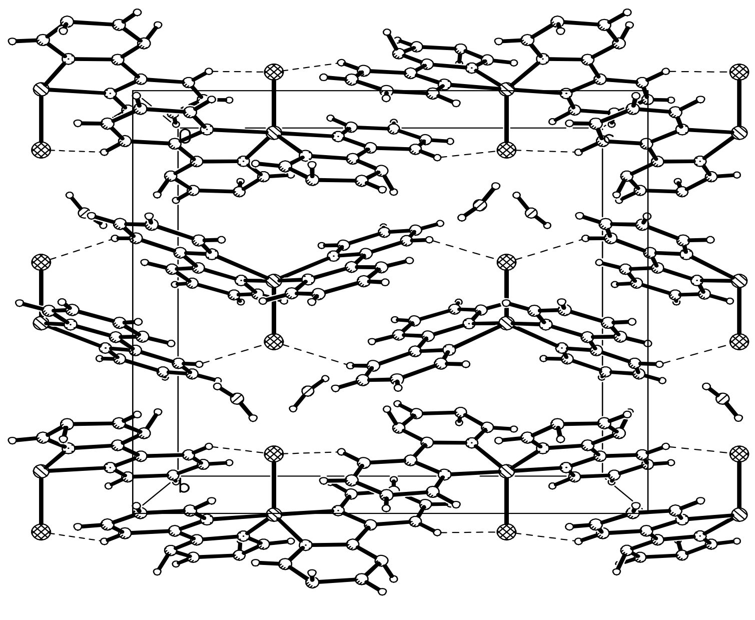

Fig. S2. The packing diagram of the complex viewed along *a* axis

S-2

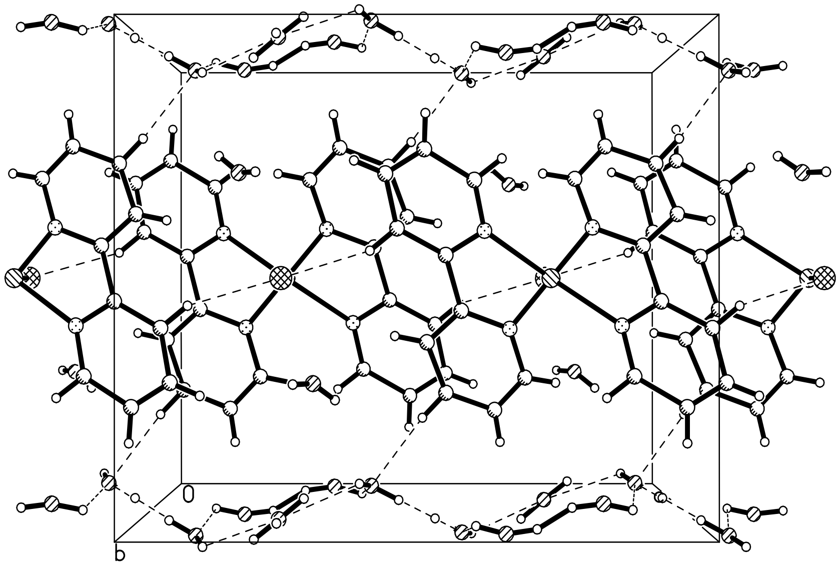

Fig. S3. The packing diagram of the complex viewed along *b* axis

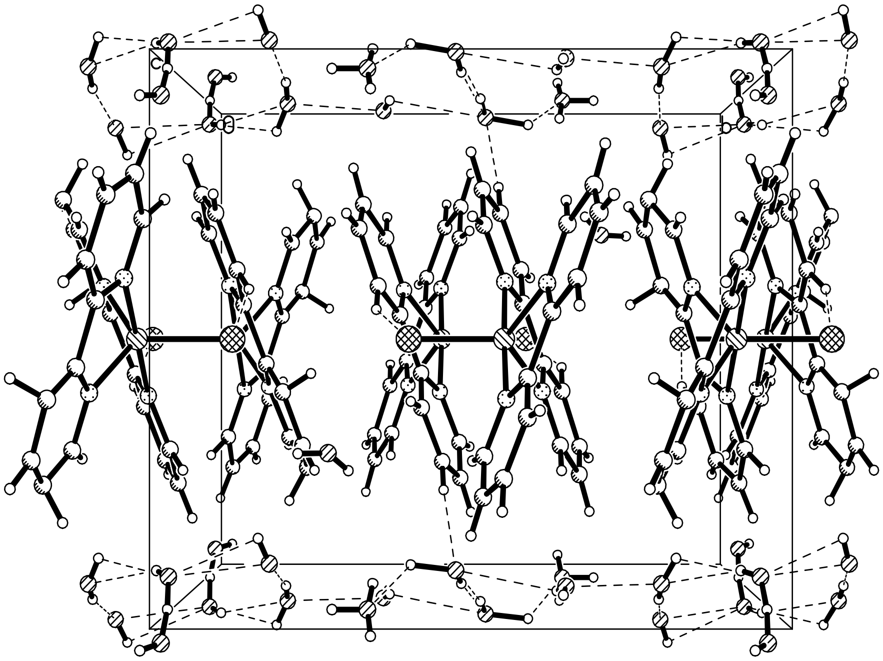

Fig. S4. The packing diagram of the complex viewed along *c* axis

S-3

3. Crystallographic data for the compound 1

Table S1. Crystal data and structure refinement

Empirical formula	C_{40} H_{56} Cl_2 Cu_2 N_8 O_{13}
Formula weight	1054.91
Temperature	293(2) K
Wavelength	0.71073 Å
Crystal system, space group	Monolinic, *P*2/c
Unit cell dimensions	a = 12.344(3)Å α= 90 deg.<br>b = 14.344(3) Å β= 90.04(3) deg.<br>c = 14.912(3) Å γ= 90 deg.
Volume	2640.4(10) Å^3
Z, Calculated density	2, 1.327 Mg/m^3
Absorption coefficient	0.969 mm^{-1}
F(000)	1096
Theta range for data collection	1.42 to 25.00 deg.
Limiting indices	-14<=h<=0,-17<=k<=0,-17<=l<=17
Reflections collected / unique	4757 / 4524 [R(int) = 0.0432]
Completeness to theta = 25.0	98.3 %
Refinement method	Full-matrix least-squares on F^2
Data / restraints / parameters	4524 / 18 / 332
Goodness-of-fit on F^2	1.200
Final R indices [I>2sigma(I)]	R1 = 0.0821, wR2 = 0.2525
R indices (all data)	R1 = 0.1516, wR2 = 0.2976
Extinction coefficient	0.0053(17)
Largest diff. peak and hole	0.965 and -0.660 e. Å^{-3}

4. Table of bond lengths and angles

Table S2. Bond lengths [Å] and angles [deg]

Cu(1)-N(2)	1.885(6)	N(2)-Cu(1)-N(2)#1	118.4(4)
Cu(1)-N(2)#1	1.885(6)	N(2)-Cu(1)-Cl(1)	120.80(1)
Cu(1)-N(1)	2.433(7)	N(2)#1-Cu(1)-Cl(1)	120.80(1)
Cu(1)-N(1)#1	2.433(7)	N(2)-Cu(1)-N(1)	83.2(3)
Cu(1)-Cl(1)	2.283(4)	N(2)#1-Cu(1)-N(1)	96.7(2)
N(1)-C(5)	1.320(9)	Cl(1)-Cu(1)-N(1)	90.20(2)
N(1)-C(1)	1.500(1)	N(2)-Cu(1)-N(1)#1	96.7(2)
N(2)-C(10)	1.500(1)	N(2)#1-Cu(1)-N(1)#1	83.2(3)
N(2)-C(6)	1.65(1)	Cl(1)-Cu(1)-N(1)#1	90.20(2)
C(1)-C(2)	1.475(9)	N(1)-Cu(1)-N(1)#1	179.7(3)
C(2)-C(3)	1.330(2)	C(5)-N(1)-C(1)	102.2(7)
C(3)-C(4)	1.560(1)	C(5)-N(1)-Cu(1)	118.2(5)
C(4)-C(5)	1.528(8)	C(1)-N(1)-Cu(1)	139.5(5)
C(5)-C(6)	1.526(8)	C(10)-N(2)-C(6)	135.7(6)
C(6)-C(7)	1.306(8)	C(10)-N(2)-Cu(1)	119.6(5)
C(7)-C(8)	1.540(2)	C(6)-N(2)-Cu(1)	104.7(4)
C(8)-C(9)	1.580(3)	N(1)-C(1)-C(2)	138.5(8)
C(9)-C(10)	1.303(8)	C(3)-C(2)-C(1)	122.9(9)
Cu(2)-N(4)#2	1.755(6)	C(2)-C(3)-C(4)	100.0(8)
Cu(2)-N(4)	1.755(6)	C(3)-C(4)-C(5)	136.1(8)
Cu(2)-Cl(2)	2.287(4)	N(1)-C(5)-C(6)	99.6(6)
Cu(2)-N(3)	2.441(8)	N(1)-C(5)-C(4)	120.3(7)
Cu(2)-N(3)#2	2.441(8)	C(6)-C(5)-C(4)	140.1(7)
N(3)-C(15)	1.255(9)	C(7)-C(6)-N(2)	111.7(7)
N(3)-C(11)	1.420(1)	C(7)-C(6)-C(5)	114.2(8)
N(4)-C(20)	1.490(2)	N(2)-C(6)-C(5)	134.1(6)
N(4)-C(16)	1.530(1)	C(6)-C(7)-C(8)	111.5(8)
C(11)-C(12)	1.630(2)	C(7)-C(8)-C(9)	136.1(7)
C(12)-C(13)	1.240(1)	C(10)-C(9)-C(8)	113.2(9)
C(13)-C(14)	1.500(1)	C(9)-C(10)-N(2)	111.5(9)
C(14)-C(15)	1.570(1)	N(4)#2-Cu(2)-N(4)	177.6(5)
C(15)-C(16)	1.590(1)	N(4)#2-Cu(2)-Cl(2)	91.2(2)
C(16)-C(17)	1.261(9)	N(4)-Cu(2)-Cl(2)	91.2(2)
C(17)-C(18)	1.560(1)	N(4)#2-Cu(2)-N(3)	96.4(3)
C(18)-C(19)	1.540(1)	N(4)-Cu(2)-N(3)	82.6(3)
C(19)-C(20)	1.230(1)	Cl(2)-Cu(2)-N(3)	113.4(2)

N(4)#2-Cu(2)-N(3)#2	82.6(3)
N(4)-Cu(2)-N(3)#2	96.4(3)
Cl(2)-Cu(2)-N(3)#2	113.2(2)
N(3)-Cu(2)-N(3)#2	133.3(3)
C(15)-N(3)-C(11)	105.6(8)
C(15)-N(3)-Cu(2)	114.8(6)
C(11)-N(3)-Cu(2)	139.5(5)
C(20)-N(4)-C(16)	132.1(6)
C(20)-N(4)-Cu(2)	117.2(6)
C(16)-N(4)-Cu(2)	110.4(5)
N(3)-C(11)-C(12)	135.3(7)
C(13)-C(12)-C(11)	120.0(1)
C(12)-C(13)-C(14)	105.0(1)
C(13)-C(14)-C(15)	134.4(7)
N(3)-C(15)-C(14)	120.2(8)
N(3)-C(15)-C(16)	102.1(8)
C(14)-C(15)-C(16)	137.7(6)
C(17)-C(16)-N(4)	117.6(9)
C(17)-C(16)-C(15)	112.6(9)
N(4)-C(16)-C(15)	129.7(6)
C(16)-C(17)-C(18)	108.3(9)
C(19)-C(18)-C(17)	133.8(7)
C(20)-C(19)-C(18)	114.0(1)
C(19)-C(20)-N(4)	114.3(1)

Symmetry code: #1 -x-1, y, -z+3/2，#2 -x+1, y, -z+1/2

S-6

5. Table of Geometrical Parameters of Hydrogen Bonds for the Water Cluster

Table S3. Geometrical Parameters of Hydrogen Bonds (Å, deg) for the Water Cluster

length		angle	
O1w-O2w	2.740(1)	O2w…O1w…O1wA	115.28
O1w-O7w	2.948(1)	O2w…O1w…O7w	130.28
O1w-C(11)[a]	3.372(1)	O1wA…O1w…O7w	113.35
O1w-C(12)[a]	3.361(1)	O1w…O2w…O3w	113.25
C(1)-O3w[a]	3.348(1)	O1w…O2w…O6wB	119.13
C(1)-O2w[a]	3.386(1)	O3w…O2w…O6wB	125.39
Cl(2)-O5w[a]	3.454(1)	O3wA…O3w…O6wC	111.78
C(12)-O2w[a]	3.396(2)	O3wA…O3w…O2w	111.75
C(18)-O7w[b]	3.264(1)	O6w…O3w…O2w	114.28
O2w-O3w	2.934	O6wD…O4w…O6wE	78.77
O2w-C(2)[a]	3.386(1)	O6wD…O4w…O7w	166.87
O3w-O6w[c]	2.625(1)	O6wE…O4w…O7wA	166.87
O5w-O6w[b]	3.176	O6wE…O4w…O7w	90.53
		O7wA…O4w…O7w	101.02
		O3wF…O6w…O5wG	111.13
		O3wF…O6w…O4wF	114.47
		O3wF…O6w…O2wB	116.41
		O5wG…O6w…O2wB	122.34
		O4wD…O6w…O2wB	112.52
		O1w…O7w…O7wD	93.04
		O1w…O7w…O4w	106.12
		O7wD…O7w…O4w	135.42
		O7w-H1…O4w	123.53
		O2w-H2…O6w	165.40
		O7w-H3…O7w	171.87
		O2w-H4…O3w	121.08
		O3w-H5…O2w	145.59
		O3w-H6…O3w	172.06
		O1w-H10…O1w	164.09

Symmetry code: a, 1-x, y, 1/2-z; b, x, 1-y, -1/2+z; c, x, -y, -1/2+z. A, -x, y, 1/2-z; B, -x, y, 3/2-z; C, x, -y, -1/2+z; D, -x, 1-y, 1-z; E, x, 1-y, -1/2+z; F, x, -y, 1/1+z;

6. Table of *π-π* Interactions (Face-to-Face) and C-*π* Interactions

Table S4. *π-π* Interactions (Face-to-Face) and C-*π* Interactions in complex 1[a]

ring(*i*)→ring(*j*)/C	distance between the (*i,j*) ring centroids(Å)	dihedral angle (*i,j*) (deg)	distance of centroid(*i*) from ring (*j*) (Å)
R1→R5^{i}	3.813	2.55	3.268
R1→R6^{ii}	4.254	56.05	0.567
R1→R7^{iii}	4.287	28.65	4.175
R2→R5^{iv}	3.813	2.55	3.268
R2→R6^{iii}	4.254	56.05	0.567
R2→R7^{ii}	4.287	28.65	4.175
R3→R6^{iii}	4.016	28.02	3.982
R4→R6^{ii}	4.016	28.02	3.982
R5→R6^{i}	3.730	1.96	3.266
R7→R5^{iii}	4.088	26.36	3.597
R7→R8^{i}	3.783	0.43	3.431
C4→R1^{i}	3.251		3.334
C4→R2^{v}	3.251		3.334

[a] Symmetry code: (i)=*1-x, -y, -z*; (ii)=*1-x, y, 1/2-z*; (iii)=*x, y, z*; (iv)=*x, -y, 1/2+z*; (v)=*x,-y,-1/2+z*. R(i)/R(j) denotes the *i*th/*j*th rings of phen: R(1)=Cu(1)/N(1)/C(5)/C(6)/N(2); R(2)=Cu(1)/N(1)a/C(5)a/C(6)a/N(2)a;R(3)=Cu(2)/N(3)/C(15)/C(16)/N(4);R(4)=Cu(2)/N(3)b/C(15)b/C(16)b/N(4)b; R(5)=N(1)/C(1)/C(2)/C(3)/C(4)/C(5); R(6)=N(2)/C(6)/C(7)/C(8)/C(9)/C(10); R(7)=N(3)/C(11)/C(12)/C(13)/C(14)/C(15);R(8)=N(4)/C(16)/C(17)/C(18)/C(19)/C(20).

7. Schematic view of the Cu-layers of the 2D network.

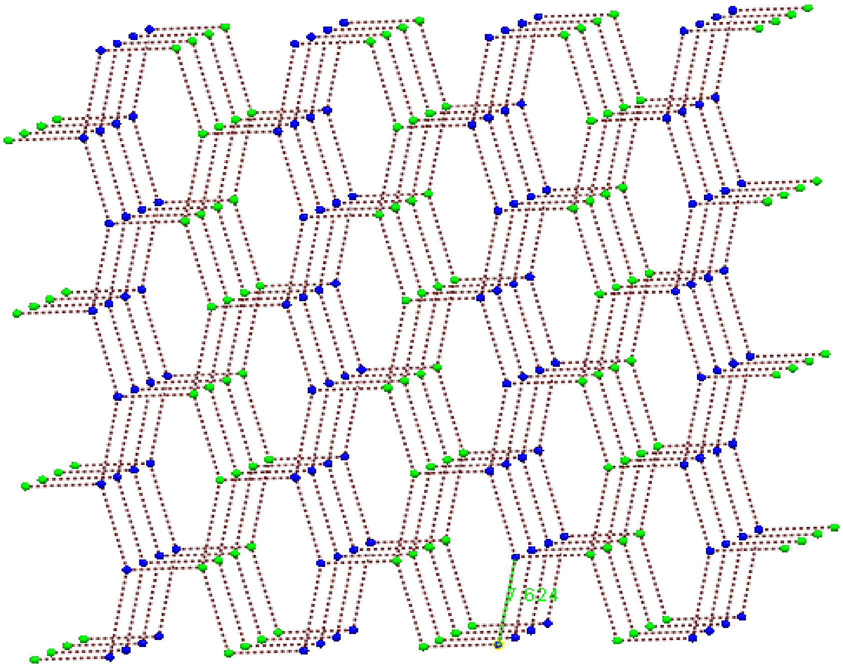

(a)

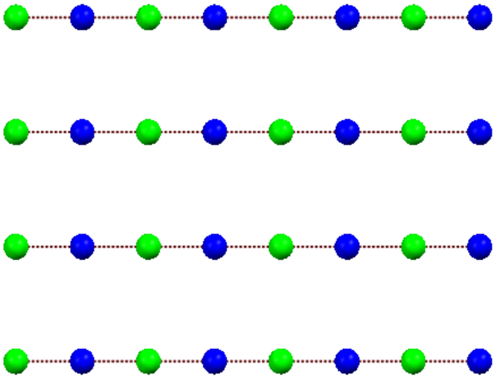

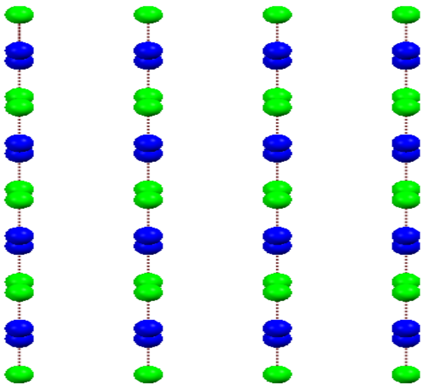

(b) **(c)**

Fig. S5. Ball and stick model of Cu atoms in compound 1 showing 2D sheet architecture. (a) along the *a* axis; (b) along the *b* axis; (c) along the *c* axis

S-9

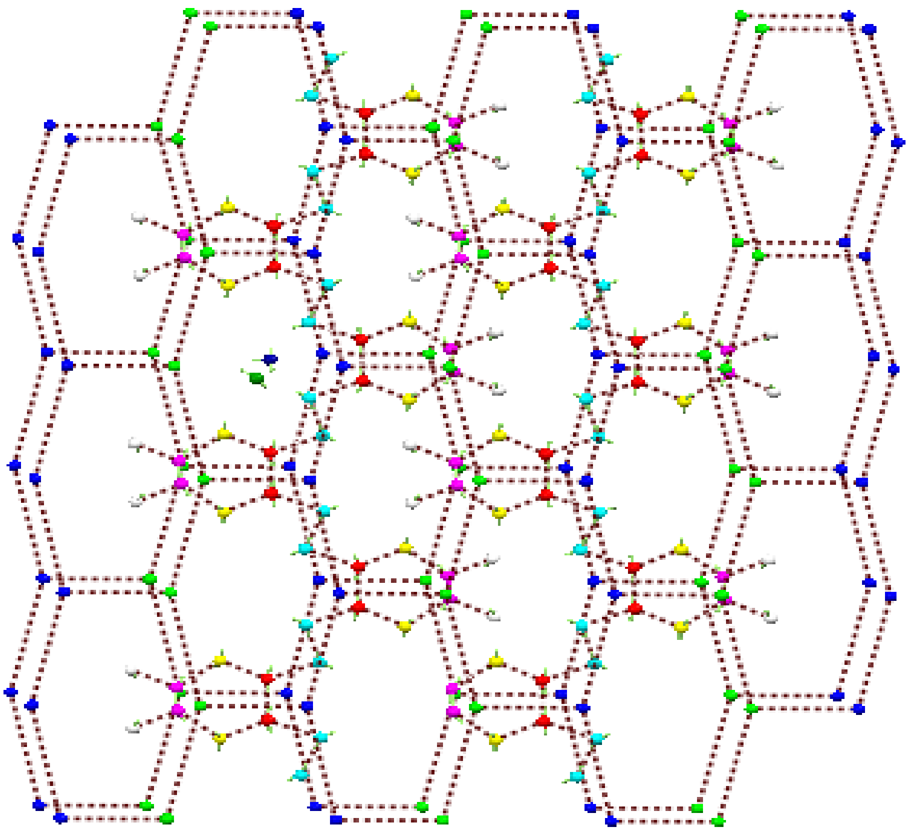

Fig. S6. **Packing of anionic water cluster and Cu-layer along the *a* axis.**

S10

8. The CIF file for $[CuCl(bipy)_2]_2[(OH)_2(H_2O)_{11}]$

```
_audit_creation_method            SHELXL-97
_chemical_name_systematic
;
 ?
;
_chemical_name_common             ?
_chemical_melting_point           ?
_chemical_formula_moiety          ?
_chemical_formula_sum
 'C80 H116 Cl4 Cu4 N16 O26'
_chemical_formula_weight          2113.85

loop_
 _atom_type_symbol
 _atom_type_description
 _atom_type_scat_dispersion_real
 _atom_type_scat_dispersion_imag
 _atom_type_scat_source
 'C'  'C'   0.0033   0.0016
 'International Tables Vol C Tables 4.2.6.8 and 6.1.1.4'
 'H'  'H'   0.0000   0.0000
 'International Tables Vol C Tables 4.2.6.8 and 6.1.1.4'
 'N'  'N'   0.0061   0.0033
 'International Tables Vol C Tables 4.2.6.8 and 6.1.1.4'
 'O'  'O'   0.0106   0.0060
 'International Tables Vol C Tables 4.2.6.8 and 6.1.1.4'
 'Cl'  'Cl'   0.1484   0.1585
 'International Tables Vol C Tables 4.2.6.8 and 6.1.1.4'
 'Cu'  'Cu'   0.3201   1.2651
 'International Tables Vol C Tables 4.2.6.8 and 6.1.1.4'

_symmetry_cell_setting            ?
_symmetry_space_group_name_H-M    ?

loop_
 _symmetry_equiv_pos_as_xyz
 'x, y, z'
 '-x, y, -z+1/2'
 '-x, -y, -z'
 'x, -y, z-1/2'
```

```
_cell_length_a                    12.344(3)
_cell_length_b                    14.344(3)
_cell_length_c                    14.912(3)
_cell_angle_alpha                 90.00
_cell_angle_beta                  90.04(3)
_cell_angle_gamma                 90.00
_cell_volume                      2640.4(10)
_cell_formula_units_Z             1
_cell_measurement_temperature     293(2)
_cell_measurement_reflns_used     ?
_cell_measurement_theta_min       ?
_cell_measurement_theta_max       ?

_exptl_crystal_description        ?
_exptl_crystal_colour             ?
_exptl_crystal_size_max           ?
_exptl_crystal_size_mid           ?
_exptl_crystal_size_min           ?
_exptl_crystal_density_meas       ?
_exptl_crystal_density_diffrn     1.329
_exptl_crystal_density_method     'not measured'
_exptl_crystal_F_000              1100
_exptl_absorpt_coefficient_mu     0.969
_exptl_absorpt_correction_type    ?
_exptl_absorpt_correction_T_min   ?
_exptl_absorpt_correction_T_max   ?
_exptl_absorpt_process_details    ?

_exptl_special_details
;
 ?
;

_diffrn_ambient_temperature       293(2)
_diffrn_radiation_wavelength      0.71073
_diffrn_radiation_type            MoK\a
_diffrn_radiation_source          'fine-focus sealed tube'
_diffrn_radiation_monochromator   graphite
_diffrn_measurement_device_type   ?
_diffrn_measurement_method        ?
_diffrn_detector_area_resol_mean  ?
_diffrn_standards_number          ?
_diffrn_standards_interval_count  ?
_diffrn_standards_interval_time   ?
```

```
_diffrn_standards_decay_%         ?
_diffrn_reflns_number             4757
_diffrn_reflns_av_R_equivalents   0.0432
_diffrn_reflns_av_sigmaI/netI     0.0512
_diffrn_reflns_limit_h_min        -14
_diffrn_reflns_limit_h_max        0
_diffrn_reflns_limit_k_min        -17
_diffrn_reflns_limit_k_max        0
_diffrn_reflns_limit_l_min        -17
_diffrn_reflns_limit_l_max        17
_diffrn_reflns_theta_min          1.42
_diffrn_reflns_theta_max          25.50
_reflns_number_total              4524
_reflns_number_gt                 2397
_reflns_threshold_expression      >2sigma(I)

_computing_data_collection        ?
_computing_cell_refinement        ?
_computing_data_reduction         ?
_computing_structure_solution     'SHELXS-97 (Sheldrick, 1990)'
_computing_structure_refinement   'SHELXL-97 (Sheldrick, 1997)'
_computing_molecular_graphics     ?
_computing_publication_material   ?

_refine_special_details
;
 Refinement of F^2^ against ALL reflections.  The weighted R-factor wR and
 goodness of fit S are based on F^2^, conventional R-factors R are based
 on F, with F set to zero for negative F^2^. The threshold expression of
 F^2^ > 2sigma(F^2^) is used only for calculating R-factors(gt) etc. and is
 not relevant to the choice of reflections for refinement.  R-factors based
 on F^2^ are statistically about twice as large as those based on F, and R-
 factors based on ALL data will be even larger.
;

_refine_ls_structure_factor_coef  Fsqd
_refine_ls_matrix_type            full
_refine_ls_weighting_scheme       calc
_refine_ls_weighting_details
 'calc w=1/[\s^2^(Fo^2^)+(0.1586P)^2^+0.6926P] where P=(Fo^2^+2Fc^2^)/3'
_atom_sites_solution_primary      direct
_atom_sites_solution_secondary    difmap
_atom_sites_solution_hydrogens    geom
_refine_ls_hydrogen_treatment     mixed
```

```
_refine_ls_extinction_method      SHELXL
_refine_ls_extinction_coef        0.0048(14)
_refine_ls_extinction_expression
 'Fc^*^=kFc[1+0.001xFc^2^\l^3^/sin(2\q)]^-1/4^'
_refine_ls_number_reflns          4524
_refine_ls_number_parameters      336
_refine_ls_number_restraints      15
_refine_ls_R_factor_all           0.1429
_refine_ls_R_factor_gt            0.0747
_refine_ls_wR_factor_ref          0.2713
_refine_ls_wR_factor_gt           0.2226
_refine_ls_goodness_of_fit_ref    1.088
_refine_ls_restrained_S_all       1.118
_refine_ls_shift/su_max           5.295
_refine_ls_shift/su_mean          0.158

loop_
 _atom_site_label
 _atom_site_type_symbol
 _atom_site_fract_x
 _atom_site_fract_y
 _atom_site_fract_z
 _atom_site_U_iso_or_equiv
 _atom_site_adp_type
 _atom_site_occupancy
 _atom_site_symmetry_multiplicity
 _atom_site_calc_flag
 _atom_site_refinement_flags
 _atom_site_disorder_assembly
 _atom_site_disorder_group
Cu1 Cu -0.5000 -0.05695(10) 0.7500 0.0628(5) Uani 1 2 d S . .
Cl1 Cl -0.5000 0.1021(2) 0.7500 0.0854(9) Uani 1 2 d S . .
N1 N -0.3914(4) -0.0578(4) 0.6142(4) 0.0605(15) Uani 1 1 d . . .
N2 N -0.5976(4) -0.1244(4) 0.6783(4) 0.0578(15) Uani 1 1 d . . .
C1 C -0.2871(6) -0.0234(6) 0.5877(5) 0.074(2) Uani 1 1 d . . .
H1A H -0.2504 0.0051 0.6348 0.089 Uiso 1 1 calc R . .
C2 C -0.2154(6) -0.0222(6) 0.4924(6) 0.078(2) Uani 1 1 d . . .
H2A H -0.1457 0.0022 0.4920 0.093 Uiso 1 1 calc R . .
C3 C -0.2561(7) -0.0547(5) 0.4209(5) 0.072(2) Uani 1 1 d . . .
H3A H -0.2239 -0.0562 0.3646 0.087 Uiso 1 1 calc R . .
C4 C -0.3654(6) -0.0897(5) 0.4448(5) 0.070(2) Uani 1 1 d . . .
H4A H -0.4040 -0.1152 0.3972 0.084 Uiso 1 1 calc R . .
C5 C -0.4312(5) -0.0912(5) 0.5430(4) 0.0587(18) Uani 1 1 d . . .
C6 C -0.5501(5) -0.1286(4) 0.5800(4) 0.0565(18) Uani 1 1 d . . .
```

C7 C -0.6046(7) -0.1635(5) 0.5188(5) 0.077(2) Uani 1 1 d . . .
H7A H -0.5674 -0.2157 0.4930 0.092 Uiso 1 1 d R . .
C8 C -0.7127(7) -0.1960(6) 0.5591(6) 0.082(3) Uani 1 1 d . . .
H8A H -0.7594 -0.2210 0.5165 0.099 Uiso 1 1 calc R . .
C9 C -0.7634(7) -0.1965(6) 0.6590(6) 0.087(3) Uani 1 1 d . . .
H9B H -0.8314 -0.1641 0.6576 0.104 Uiso 1 1 d R . .
C10 C -0.7037(6) -0.1590(5) 0.7168(5) 0.071(2) Uani 1 1 d . . .
H10A H -0.7226 -0.1530 0.7769 0.086 Uiso 1 1 calc R . .
Cu2 Cu 0.5000 0.44466(10) 0.2500 0.0722(5) Uani 1 2 d S . .
Cl2 Cl 0.5000 0.6039(2) 0.2500 0.0911(10) Uani 1 2 d S . .
N3 N 0.5999(5) 0.3772(4) 0.1241(4) 0.0670(17) Uani 1 1 d . . .
N4 N 0.3918(5) 0.4424(4) 0.1730(4) 0.0715(18) Uani 1 1 d . . .
C11 C 0.7044(6) 0.3452(5) 0.1047(6) 0.077(2) Uani 1 1 d . . .
H11A H 0.7493 0.3448 0.1548 0.092 Uiso 1 1 calc R . .
C12 C 0.7661(7) 0.3081(5) 0.0137(6) 0.083(3) Uani 1 1 d . . .
H12A H 0.8384 0.2903 0.0158 0.099 Uiso 1 1 calc R . .
C13 C 0.7155(7) 0.3044(5) -0.0568(6) 0.083(3) Uani 1 1 d . . .
H13A H 0.7418 0.2853 -0.1123 0.100 Uiso 1 1 calc R . .
C14 C 0.6039(6) 0.3363(5) -0.0359(6) 0.072(2) Uani 1 1 d . . .
H14A H 0.5573 0.3347 -0.0850 0.087 Uiso 1 1 calc R . .
C15 C 0.5488(6) 0.3722(5) 0.0528(5) 0.064(2) Uani 1 1 d . . .
C16 C 0.4311(6) 0.4097(5) 0.0822(5) 0.065(2) Uani 1 1 d . . .
C17 C 0.3641(7) 0.4116(5) 0.0185(6) 0.083(3) Uani 1 1 d . . .
H17A H 0.3782 0.3940 -0.0404 0.099 Uiso 1 1 calc R . .
C18 C 0.2554(6) 0.4478(5) 0.0541(6) 0.088(3) Uani 1 1 d . . .
H18A H 0.2018 0.4506 0.0103 0.105 Uiso 1 1 calc R . .
C19 C 0.2162(6) 0.4804(6) 0.1460(6) 0.081(3) Uani 1 1 d . . .
H19A H 0.1457 0.5000 0.1572 0.097 Uiso 1 1 calc R . .
C20 C 0.2864(6) 0.4781(6) 0.2030(6) 0.086(3) Uani 1 1 d . . .
H20A H 0.2743 0.4975 0.2616 0.103 Uiso 1 1 calc R . .
O1W O 0.0304(8) 0.3382(6) 0.3409(9) 0.145(4) Uani 1 1 d D . .
O2W O 0.0262(9) 0.1678(7) 0.4218(10) 0.152(4) Uani 1 1 d D . .
O3W O 0.0550(7) 0.0108(8) 0.2971(7) 0.211(6) Uani 1 1 d D . .
O7W O 0.0534(10) 0.5271(12) 0.4151(11) 0.276(10) Uani 1 1 d D . .
O6W O -0.0670(15) 0.1528(13) 0.6234(14) 0.303(8) Uiso 1 1 d D . .
H13 H 0.006(18) 0.15(3) 0.62(3) 2.0(12) Uiso 1 1 d D . .
H14 H -0.122(3) 0.142(4) 0.656(3) 0.049(17) Uiso 1 1 d D . .
O5W O 0.286(2) 0.7415(14) 0.316(2) 0.413(15) Uiso 1 1 d D . .
O4W O 0.0000 0.677(3) 0.2500 0.44(2) Uiso 1 2 d SD . .
H6 H 0.0000 0.006(4) 0.2500 0.000(14) Uiso 1 2 d SD . .
H5 H 0.060(3) 0.038(2) 0.3455(15) 0.000(10) Uiso 1 1 d D . .
H4 H 0.080(6) 0.146(7) 0.392(6) 0.11(4) Uiso 1 1 d D . .
H3 H 0.020(4) 0.514(3) 0.465(2) 0.000(10) Uiso 1 1 d D . .
H1 H 0.091(8) 0.576(4) 0.392(7) 0.15(4) Uiso 1 1 d D . .

```
H8 H 0.250(4) 0.709(3) 0.354(3) 0.028(13) Uiso 1 1 d D . .
H9 H 0.00(3) 0.389(14) 0.35(3) 0.8(3) Uiso 1 1 d D . .
H2 H -0.010(5) 0.167(5) 0.471(3) 0.030(18) Uiso 1 1 d D . .
H7 H 0.290(5) 0.789(3) 0.283(4) 0.069(19) Uiso 1 1 d D . .
H10 H 0.028(8) 0.346(6) 0.2842(13) 0.09(4) Uiso 1 1 d D . .
H11 H 0.042(5) 0.666(6) 0.294(3) 0.09(3) Uiso 1 1 d D . .
H12 H 0.0000 0.737(3) 0.2500 0.07(3) Uiso 1 2 d SD . .

loop_
 _atom_site_aniso_label
 _atom_site_aniso_U_11
 _atom_site_aniso_U_22
 _atom_site_aniso_U_33
 _atom_site_aniso_U_23
 _atom_site_aniso_U_13
 _atom_site_aniso_U_12
Cu1 0.0541(7) 0.0833(10) 0.0509(7) 0.000 -0.0193(5) 0.000
Cl1 0.129(3) 0.0716(19) 0.0552(15) 0.000 -0.0341(16) 0.000
N1 0.055(3) 0.071(4) 0.055(3) 0.002(3) -0.019(3) 0.000(3)
N2 0.058(3) 0.057(3) 0.058(3) 0.001(3) -0.023(3) 0.001(3)
C1 0.064(5) 0.082(6) 0.077(5) 0.001(4) -0.023(4) -0.003(4)
C2 0.069(5) 0.083(6) 0.081(6) 0.015(5) -0.017(4) -0.002(4)
C3 0.084(5) 0.072(5) 0.061(5) 0.015(4) 0.006(4) 0.021(4)
C4 0.087(6) 0.062(5) 0.062(5) 0.010(4) -0.026(4) 0.007(4)
C5 0.069(4) 0.054(4) 0.053(4) 0.006(3) -0.024(3) 0.016(3)
C6 0.068(4) 0.040(4) 0.062(4) 0.002(3) -0.028(4) 0.006(3)
C7 0.096(6) 0.046(4) 0.088(6) -0.007(4) -0.049(5) 0.012(4)
C8 0.090(6) 0.064(5) 0.093(6) -0.007(4) -0.052(5) 0.002(4)
C9 0.080(5) 0.064(5) 0.116(7) -0.001(5) -0.059(5) -0.001(4)
C10 0.072(5) 0.071(5) 0.071(5) 0.006(4) -0.029(4) -0.003(4)
Cu2 0.0641(8) 0.0776(9) 0.0749(9) 0.000 -0.0449(7) 0.000
Cl2 0.121(2) 0.0679(18) 0.0845(19) 0.000 -0.0634(18) 0.000
N3 0.072(4) 0.053(3) 0.076(4) 0.008(3) -0.046(3) -0.003(3)
N4 0.068(4) 0.064(4) 0.082(4) 0.007(3) -0.044(3) -0.005(3)
C11 0.075(5) 0.063(5) 0.092(6) -0.002(4) -0.044(4) 0.008(4)
C12 0.084(5) 0.063(5) 0.101(6) 0.003(5) -0.046(5) 0.006(4)
C13 0.115(7) 0.056(5) 0.079(5) 0.003(4) -0.036(5) -0.007(5)
C14 0.080(5) 0.054(4) 0.083(5) 0.003(4) -0.037(4) -0.007(4)
C15 0.077(5) 0.047(4) 0.068(5) 0.010(4) -0.042(4) -0.009(4)
C16 0.070(5) 0.049(4) 0.078(5) 0.012(3) -0.053(4) -0.012(3)
C17 0.102(6) 0.062(5) 0.085(5) 0.021(4) -0.062(5) -0.015(4)
C18 0.072(5) 0.072(5) 0.118(7) 0.037(5) -0.068(5) -0.014(4)
C19 0.068(5) 0.082(6) 0.093(6) 0.021(5) -0.044(5) -0.008(4)
C20 0.069(5) 0.086(6) 0.103(6) 0.005(5) -0.047(5) -0.001(4)
```

O1W 0.134(6) 0.099(6) 0.201(11) -0.002(6) -0.093(7) 0.021(4)
O2W 0.125(10) 0.129(9) 0.201(14) 0.002(10) -0.059(9) -0.017(8)
O3W 0.148(9) 0.237(12) 0.250(15) -0.129(11) 0.041(9) -0.082(8)
O7W 0.155(13) 0.298(17) 0.38(2) -0.18(2) -0.180(15) 0.062(13)

_geom_special_details ;
All esds (except the esd in the dihedral angle between two l.s. planes)
are estimated using the full covariance matrix. The cell esds are taken
into account individually in the estimation of esds in distances, angles
and torsion angles; correlations between esds in cell parameters are only
used when they are defined by crystal symmetry. An approximate (isotropic)
treatment of cell esds is used for estimating esds involving l.s. planes. ;

loop_
_geom_bond_atom_site_label_1
_geom_bond_atom_site_label_2
_geom_bond_distance
_geom_bond_site_symmetry_2
_geom_bond_publ_flag
Cu1 N2 1.879(5) . ?
Cu1 N2 1.879(5) 2_456 ?
Cu1 Cl1 2.281(3) . ?
Cu1 N1 2.430(6) 2_456 ?
Cu1 N1 2.430(6) . ?
N1 C5 1.264(8) . ?
N1 C1 1.435(9) . ?
N2 C10 1.514(9) . ?
N2 C6 1.580(9) . ?
C1 C2 1.674(12) . ?
C2 C3 1.267(11) . ?
C3 C4 1.483(10) . ?
C4 C5 1.675(10) . ?
C5 C6 1.657(10) . ?
C6 C7 1.239(9) . ?
C7 C8 1.536(12) . ?
C8 C9 1.617(13) . ?
C9 C10 1.255(9) . ?
Cu2 N4 1.761(5) 2_655 ?
Cu2 N4 1.761(5) . ?
Cu2 Cl2 2.284(3) . ?
Cu2 N3 2.446(7) . ?
Cu2 N3 2.446(7) 2_655 ?
N3 C15 1.239(7) . ?
N3 C11 1.399(9) . ?

N4 C20 1.468(11) . ?
N4 C16 1.513(10) . ?
C11 C12 1.645(12) . ?
C12 C13 1.223(10) . ?
C13 C14 1.485(12) . ?
C14 C15 1.574(11) . ?
C15 C16 1.610(11) . ?
C16 C17 1.260(8) . ?
C17 C18 1.534(12) . ?
C18 C19 1.527(13) . ?
C19 C20 1.214(9) . ?

loop_
 _geom_angle_atom_site_label_1
 _geom_angle_atom_site_label_2
 _geom_angle_atom_site_label_3
 _geom_angle
 _geom_angle_site_symmetry_1
 _geom_angle_site_symmetry_3
 _geom_angle_publ_flag
N2 Cu1 N2 118.0(3) . 2_456 ?
N2 Cu1 Cl1 121.00(17) . . ?
N2 Cu1 Cl1 121.00(17) 2_456 . ?
N2 Cu1 N1 96.8(2) . 2_456 ?
N2 Cu1 N1 82.9(2) 2_456 2_456 ?
Cl1 Cu1 N1 90.27(14) . 2_456 ?
N2 Cu1 N1 82.9(2) . . ?
N2 Cu1 N1 96.8(2) 2_456 . ?
Cl1 Cu1 N1 90.27(14) . . ?
N1 Cu1 N1 179.5(3) 2_456 . ?
C5 N1 C1 104.4(6) . . ?
C5 N1 Cu1 119.2(5) . . ?
C1 N1 Cu1 136.3(5) . . ?
C10 N2 C6 131.4(5) . . ?
C10 N2 Cu1 120.5(4) . . ?
C6 N2 Cu1 108.0(4) . . ?
N1 C1 C2 135.4(7) . . ?
C3 C2 C1 120.1(7) . . ?
C2 C3 C4 106.3(7) . . ?
C3 C4 C5 131.1(6) . . ?
N1 C5 C6 100.8(6) . . ?
N1 C5 C4 122.7(6) . . ?
C6 C5 C4 136.5(6) . . ?
C7 C6 N2 119.8(7) . . ?

C7 C6 C5 111.5(7) . . ?
N2 C6 C5 128.8(5) . . ?
C6 C7 C8 107.8(8) . . ?
C7 C8 C9 134.4(6) . . ?
C10 C9 C8 113.7(8) . . ?
C9 C10 N2 112.8(8) . . ?
N4 Cu2 N4 177.9(4) 2_655 . ?
N4 Cu2 Cl2 91.1(2) 2_655 . ?
N4 Cu2 Cl2 91.1(2) . . ?
N4 Cu2 N3 96.4(2) 2_655 . ?
N4 Cu2 N3 82.8(3) . . ?
Cl2 Cu2 N3 113.29(14) . . ?
N4 Cu2 N3 82.8(3) 2_655 2_655 ?
N4 Cu2 N3 96.4(2) . 2_655 ?
Cl2 Cu2 N3 113.29(14) . 2_655 ?
N3 Cu2 N3 133.4(3) . 2_655 ?
C15 N3 C11 105.8(7) . . ?
C15 N3 Cu2 115.2(5) . . ?
C11 N3 Cu2 138.9(4) . . ?
C20 N4 C16 131.8(5) . . ?
C20 N4 Cu2 117.8(5) . . ?
C16 N4 Cu2 110.2(4) . . ?
N3 C11 C12 134.7(6) . . ?
C13 C12 C11 119.1(8) . . ?
C12 C13 C14 106.2(9) . . ?
C13 C14 C15 132.7(6) . . ?
N3 C15 C14 121.4(7) . . ?
N3 C15 C16 101.8(7) . . ?
C14 C15 C16 136.8(5) . . ?
C17 C16 N4 117.2(8) . . ?
C17 C16 C15 113.2(8) . . ?
N4 C16 C15 129.6(5) . . ?
C16 C17 C18 108.7(8) . . ?
C19 C18 C17 133.8(6) . . ?
C20 C19 C18 113.2(8) . . ?
C19 C20 N4 115.4(9) . . ?

_diffrn_measured_fraction_theta_max 0.923
_diffrn_reflns_theta_full 25.50
_diffrn_measured_fraction_theta_full 0.923
_refine_diff_density_max 0.916
_refine_diff_density_min -0.673
_refine_diff_density_rms 0.096
